# Neuroendocrine Regulation of Tumor-Associated Immune Cells

**DOI:** 10.3389/fonc.2019.01077

**Published:** 2019-10-29

**Authors:** Claudia B. Colon-Echevarria, Rocio Lamboy-Caraballo, Alexandra N. Aquino-Acevedo, Guillermo N. Armaiz-Pena

**Affiliations:** ^1^Division of Pharmacology, Department of Basic Sciences, School of Medicine, Ponce Health Sciences University, Ponce, PR, United States; ^2^Divisions of Cancer Biology and Women's Health, Ponce Research Institute, Ponce, PR, United States

**Keywords:** cancer biology, stress hormones, inflammation, immune cells, tumor microenvironment

## Abstract

Mounting preclinical and clinical evidence continues to support a role for the neuroendocrine system in the modulation of tumor biology and progression. Several studies have shown data supporting a link between chronic stress and cancer progression. Dysregulation of the sympathetic nervous system (SNS) and the hypothalamic-pituitary-adrenal (HPA) axis has been implicated in promoting angiogenesis, tumor cell proliferation and survival, alteration of the immune response and exacerbating inflammatory networks in the tumor microenvironment. Here, we review how SNS and HPA dysregulation contributes to disturbances in immune cell populations, modifies cancer biology, and impacts immunotherapy response. We also highlight several interventions aimed at circumventing the adverse effects stress has on cancer patients.

## Introduction

The stress response is a biological process mainly mediated by the sympathetic nervous system (SNS) and the hypothalamic-pituitary-adrenal (HPA) axis. Activation of the SNS and HPA axis leads to the release of neurotransmitters and hormones, such as catecholamines and glucocorticoids. These molecules mediate many biochemical and physiologic changes (e.g., increased heart rate, rapid breathing, perspiration). Moreover, catecholamines released upon activation of the SNS, such as epinephrine (EPI) and norepinephrine (NE), are capable of binding to receptors on or within immune cells through activation of α and β adrenergic receptors (ARs) (1). Correspondingly, HPA activation leads to increased glucocorticoid release and activation of glucocorticoid receptors (GR).

Prolonged activation of the SNS and HPA axis, also known as chronic stress (occurring over weeks or years), increases the exposure of the immune system to elevated stress hormone levels. The increased exposure to stress hormones disrupts physiological homeostasis and could serve as a risk factor for the development and progression of many diseases, including cancer (2–4). In contrast to acute stress, which lasts several minutes to hours and is associated with enhanced immunity and resistance to cancer, chronic stress is known to impair immune response and promote tumor growth (5, 6). Multiple studies have correlated stress-induced biobehavioral abnormalities, such as anxiety, depression and chronic stress, with tumor growth, progression, and metastasis (7–11).

A dysregulated HPA or dysfunctional SNS activity due to chronic stress warrants further attention as alterations in these systems can affect immune function in the tumor and ultimately impact its biology and disease outcome (Table 1). One of the primary mechanisms by which chronic stress affects cancer progression is through alterations in humoral and cell-mediated immunity. Chronic stress can lead to variations in the proliferative capacity of lymphocytes and suppression of immune activity relevant to cell-mediated immunity [i.e., natural killer (NK) cells, cytotoxic T-lymphocytes (CTLs), dendritic cells, macrophages] (52–54). Likewise, stress-induced immune alterations can enhance the generation and migration of many pro-inflammatory factors, such as interleukin (IL)-6, tumor necrosis factor (TNF-α) in the tumor microenvironment (6, 15, 21–23, 52). Stress-induced pro-tumoral factors, such as matrix metalloproteases (MMPs) and vascular endothelial growth factor (VEGF), can significantly influence cancer progression by stimulating tumor cell survival, tumor growth, metastasis, and evasion of the immune system (41, 55–57).

**Table 1:** Neuroendocrine regulation of immune cell function in cancer.

**Immune cell**	**Effects of HPA and/or SNS activation on immune cell function**	**Selected references**
T cells	Suppresses APCs and T_H_1 cells promoting T_H_2 cytokine production Alters distribution, proliferation and apoptosis Promotes immunosuppression and tumor progression by increasing T regulatory cell activity Decreases infiltration into tumors Reduces effectiveness of T-cell targeted immunotherapy by suppressing antitumor CD8+ T	(12–14) (15–18) (19) (20–23) (24)
Natural Killer cells	Forced swim and administration of Epi or corticosterone inhibited NK cell activity in rats Elevated levels of stress or depression were linked to decreased NK cell levels and activity, impaired cytotoxicity and altered membrane receptor expression	(25) (16, 18, 22, 26–28)
B cells	Anxious behavior in mice was associated with increased B-regulatory cell levels and tumor progression Breast cancer patients that underwent a mastectomy with high levels of stress exhibited decreased T-cell values (cellular immunity) while B-cell values were unaffected (humoral immunity)	(21) (29)
Dendritic cells	GCs induce apoptosis, represses activation, migration and promotes tolerogenic phenotypes Dual effect on migration by adrenergic stimulation Modulates the efficacy of cancer vaccines that use tumor antigen loaded DCs Inhibits DCs IL-12 production Immobilization stress in mice lead to induction of VEGF which can lead to DC maturation Exposure to chronic cold (stressor) temperature was associated to repressed activation of DCs	(30–32) (33, 34) (35) (3) (7) (36)
Myeloid-derived suppressive cells	Stimulates immune-suppressive activity High levels of stress correlated with increased levels of MDSCs in breast cancer patients Chronically stressed mice exhibited increased infiltration into tumor sites and enhanced suppressive activity toward proliferating T cells	(37, 38) (39) (19, 40)
Granulocytic myeloid derived cells	Influences generation, activity and migration toward the tumor microenvironment Chronically stressed mice displayed decreased phagocytic activity in neutrophils	(41) (42)
Macrophages	Contributes to tumor invasiveness by stimulating TAMs to increase gene expression of *mmp2, mmp9, mmp12*, and *ctsl* proteases Promotes transformation from M1 to M2 phenotype Adrenergic activation increased macrophage infiltration into tumor leading to progression Catecholamines stimulate macrophage production of pro-inflammatory cytokines High levels of stress were associated to TAM derived MMP9 Characterization of adrenergic regulated macrophages	(43) (44, 45) (10, 45) (46–49) (50) (45, 51)

*APC, antigen-presenting cell; DC, dendritic cell; T_H_, T-helper cell; GC, glucocorticoid; DC, dendritic cell; IL, interleukin; VEGF, vascular endothelial growth factor; MDSC, myeloid-derived suppressive cell; NK, natural killer cell; TAM, tumor-associated macrophage; Mmp, matrix metalloproteinases; Ctsl, cathepsin*.

Another key player in tumor-associated immunity is the lymphatic system. In cancer, the lymphatic system is a rich source of chemokines that provides a route for tumor cell escape while enhancing their invasive properties (58). In an orthotopic model of breast cancer, it was shown that chronic stress leads to lymphatic vasculature reorganization in tumors (59). Thus, stress may also enhance cancer progression and provide a route of tumor cell dissemination through the lymphatic vasculature.

**Figure 1:**
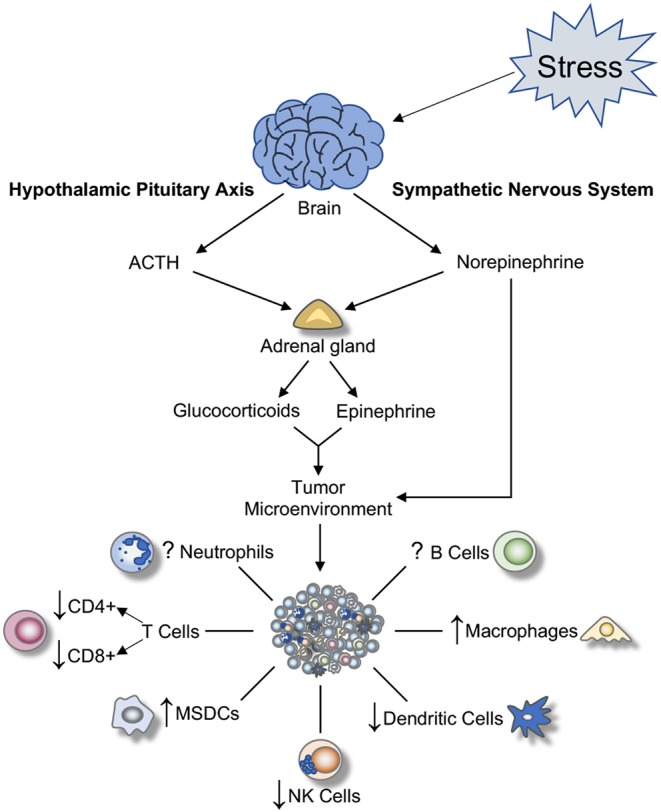
Effect of SNS and HPA activation on tumor-associated immune cells. Once a stressor is perceived, a cascade of biochemical reactions is initiated. These result in the activation of the hypothalamic-pituitary-adrenal (HPA) axis, the sympathetic nervous system (SNS), and subsequent release of hormones and glucocorticoids from adrenal glands and nerve terminals in the periphery. These hormones alter the tumor microenvironment by promoting inflammation and modulating immune cell functions within the tumor. ACTH, adrenocorticotropic hormone; NK, natural killer; MSDCs, myeloid-derived suppressive cells.

Although the effects of chronic stress on tumor cells can vary due to stress mediators released and cancer types, there are common pathological effects that arise from prolonged activation of SNS and HPA axis that contribute to cancer progression. Therefore, this review provides an overview of tumor-associated immunity while summarizing components that can be influenced by the stress response (see Figure 1). Finally, we discuss interventions for alleviating the effects of chronic stress on cancer patients and possible avenues for future research.

## Myeloid Cells

### Macrophages

Macrophages are specialized immune cells originating from circulating monocytes and capable of modulating their activity on environmental cues. In tumors, their primary function as scavengers, antigen-presenting cells (APCs) and wound healing are altered and utilized by tumor cells to survive in the host environment.

Macrophages exist in a variety of activation states and can be polarized toward functional subclasses. According to the binary polarization model, there are two primary polarization states M1 and M2 (60). Classically activated macrophages, M1, are activated by IFN-γ, lipopolysaccharide (LPS), and toll-like receptor ligands, produce pro-inflammatory and immunostimulatory cytokines and are involved in T_H_1 responses. Alternatively activated, M2, are immunosuppressive and are involved in scavenging for cellular remains, tissue repair and remodeling, and tumor progression. Tumor-associated macrophages (TAMs) are the most abundant immune cell and a vital component of the tumor microenvironment that influences multiple aspects of tumor cell biology.

Interestingly, signals from tumor cells can cause functional alterations in macrophages that can deviate them from the dualistic M1/M2-polarized definition (61). This effect provides evidence that surrounding stimuli can produce TAMs with heterogeneous phenotypes and functions (62). Many studies have evaluated the role of TAMs in the promotion of tumor growth, metastasis, angiogenesis, and maintenance of an immunosuppressive microenvironment. Immune cells and tumor cells both influence and mediate inflammation surrounding the tumor microenvironment.

TAMs promote inflammatory processes at the tumor microenvironment by accelerating angiogenesis, inducing migration and invasion of tumor cells, and promoting epithelial-mesenchymal transition (EMT) (63). The primary purpose of tumor-associated angiogenesis, the process of creating new vasculature, is to provide cancer cells with nutrients, oxygen, and routes to metastasize. TAMs support this process by secreting proangiogenic factors such as VEGF, and PDGF in the tumor microenvironment. MMP secretion by TAMs serves a dual role in tumor progression, promoting both angiogenesis and metastasis. MMP9 and MMP2 are involved in the induction of the angiogenic switch (64), while an MMP9/VEGF axis controls cancer cell intravasation (65).

Furthermore, MMPs remodel the extracellular matrix (ECM) by degrading ECM proteins such as collagen, and fibronectin (66) disrupting the basement membrane and allowing cancer cells to invade into the adjacent stroma. TAMs are also involved in promoting EMT, where cells change their phenotype to migratory mesenchymal cells and lose cell-cell adhesion proteins and markers. For instance, M2 TAMs promoted EMT in pancreatic cancer cells through the TLR4/IL-10 pathway (67), while TAM-secreted IL-8 activated the JAK2/STAT3/Snail axis in hepatocellular carcinoma (68).

For many types of solid tumors, high levels of intra-tumoral macrophage infiltration have been associated with poor prognosis (69). In patients with breast carcinoma, macrophage infiltration has been associated with high grade, lack of hormone-receptor expression, basal-like subtype, and poor outcome (70). Furthermore, in ovarian cancer patients, increased macrophage infiltration was associated with decreased overall survival (10). Also, increased macrophage density strongly correlated with poor prognosis in renal cell carcinoma, lung, and hepatocellular carcinoma (71). In addition to poor prognosis, TAM infiltration can affect chemotherapy response in esophageal cancer (72).

Other studies have demonstrated a role for activation of the adrenergic machinery on TAMs in the context of cancer progression. Lamkin et al. investigated how β-adrenergic regulation influenced macrophage polarization and phenotype (45). Interestingly, adrenergic-stimulated macrophages did not fit into one clear category of the M1-M2 binary spectrum but had increased expression of genes involved in M2 macrophage polarization. More importantly, β2-adrenergic receptors mediated the M2 promoting effects and not β1 or β3 receptors. A recent study found C/EBPβ signaling mediates the regulation of the M2 transcriptome between β-adrenergic stimulation through expression of Arg1 (51). In malignant melanoma cell lines with high reactive oxygen species (ROS), TAMs increased gene expression of *mmp2, mmp9, mmp12*, and *ctsl* proteases known to contribute to tumor invasiveness (43). Invasiveness was enhanced by TNF-α TAM secretion. 4T1 mammary carcinoma cells cultured in EPI-treated RAW 264.7 supernatant displayed increased migration and wound-healing (44). Interestingly, this same study found that EPI promoted the transformation of macrophages from M1 to an M2 phenotype. Furthermore, another study showed that NE increased expression of M2 phenotype and pro-metastatic genes in bone marrow-derived macrophages (45). This same study demonstrated that *in vivo* activation of the β-adrenergic system increased macrophage infiltration into breast cancer tumor parenchyma and triggered a metastatic cascade that resulted in distant tissue metastasis.

Dysregulation of the SNS can exacerbate tumor-promoting characteristics of TAMs. Stress and depression may cause tumor cells to increase the secretion of pro-inflammatory cytokines. For example, after NE stimulation, ovarian cancer cells produced higher levels of IL-6 (8). Catecholamines have been shown to promote macrophage secretion of pro-inflammatory cytokines such as IL-1β and TNF-α, and this might be due to surface expression of alpha and beta receptors (46). In cancer patients, studies have shown that behavioral factors can affect the tumor microenvironment and aid tumor progression. Ovarian cancer patients with high levels of stress, social isolation, and depression demonstrated increased MMP9 production by TAMs (50). Transcriptional pathways regulating inflammation are also influenced by behavioral dysregulation. Bower et al. (73) recently found that breast cancer patients reporting more social isolation exhibited upregulation of genes related to M2 polarization and EMT. Moreover, inadequate inflammatory control, impaired transcription of glucocorticoid response genes, and leukocytes with increased activity in pro-inflammatory transcription were seen in socially isolated adults (74). These studies indicate that stress hormones directly impact tumor cells and TAM while enhancing tumor growth and impairing immune function.

### Dendritic Cells

Dendritic cells (DCs) are a heterogeneous group of APCs that generate antitumor immune responses by stimulating the activation of CD4+ T-cells, CD8+ T-cells, and B-cells (75, 76). Cancer cells can modulate DC activity and promote one of its characteristic hallmarks: evasion of the immune system.

Due to their essential role in capturing, processing, and presenting antigens to T-cells, DCs have been extensively utilized in cancer immunotherapeutic strategies (77). The usual role and function of DCs can be influenced by SNS activation. For instance, glucocorticoids can induce DC apoptosis, suppress DC activation and migration, and promote a tolerogenic DC phenotype (32). Moreover, adrenergic stimulation of DCs may have opposing effects on their migration capacity either by acting as a chemotactic factor and increasing migration (mainly mediated by α1-ARs) (33) or by suppressing DC migration mainly through modulation of IL-10 and IL-12 production (mediated by β2-ARs) (34). Also, it has been noted that activation of β2-ARs can modulate cancer vaccine efficacy that utilize tumor antigen-loaded DCs, either by boosting antitumor responses or by inducing tolerance, depending on the maturation state of transferred DCs (35). Stress hormones can also inhibit the production of IL-12 in APCs like DCs, leading to reduced T_H_1 responses and stimulation of T_H_2 responses (3).

Additionally, a study that utilized orthotopic mice models of ovarian cancer found that immobilization stress stimulated tumor production of VEGF, leading to increased tumor burden (7). Importantly, VEGF produced by tumors may also promote tumor evasion of the immune system by modulating functional DCs maturation (78). For example, in a chronic cold stress mice model, it was shown that even though stressed tumor-bearing mice had more DCs, mice that were housed at normal temperatures developed more efficient APC suggesting that stress may reduce DCs activation (36).

A study in ovarian cancer patients found that depressed and anxious mood was correlated with elevated tumor stimulated T_H_2 cytokine response (23). It is known that T_H_2 cytokine responses predominantly stimulate the humoral immune response through IL-4, leading to a reduction in the cytotoxic immune response and sequential tumor growth (79, 80). Under those circumstances, IL-4 is known to block the differentiation of dendritic cells stimulated by cytotoxic precursors (81). In summary, mounting data indicate that SNS plays a significant role in suppressing the immunogenic activity of DCs.

### Neutrophils

Neutrophils represent the most abundant infiltrating white blood cell and are mainly involved in immune surveillance and inflammatory responses. The link between neutrophils and the neuroendocrine system has been well-studied (82–84). However, to the best of our knowledge, there is a gap of knowledge regarding the effects of stress on the neutrophilic response in cancer. Notably, an *in vivo* social stress mice model found that mice that established dominant/submissive relationships had different immunogenic profiles. Submissive mice (comparable to mice undergoing chronic social stress) when compared to dominant male mice, exhibited suppressed neutrophil phagocytic activity and increased melanoma metastasis (42). Interestingly, in a non-cancer setting, the effects of psychological stress on neutrophils are suggested to be dependent on the duration (acute or chronic) and type (i.e., expected or unexpected) of stress. For instance, academic examinations enhance the phagocytic activity of neutrophils while chronic stress suppresses superoxide production leading to decreased phagocytic activity (83).

### Myeloid-Derived Suppressive Cells

Myeloid-derived suppressive cells (MDSCs) are immature myeloid cells with a potent immune-suppressive activity that plays a significant role in cancer immunology. Several studies have found an accumulation of MDSCs in tumors from murine models and cancer patients (39, 85). Several tumor-derived factors (i.e., STATS, C.EBPb, NF-kB, prostaglandin E2) produce the necessary signals for the generation, accumulation, and migration of MDSCs to the tumor microenvironment (86). Because stress responses are known to elevate many of these tumor-derived factors, stress may also stimulate the immune-suppressive activity of MDSCs and promote tumor progression (37, 38). For example, breast cancer patients reporting high levels of stress (based on an Impact of Event Scale questionnaire) had elevated levels of MDSCs (when compared to low-stress patients) (39) Moreover, studies on chronically stressed mice suggest a strong correlation between stress and MDSCs suppression. Specifically, chronically stressed mice exhibited greater tumor burden associated with the infiltration of MDSCs (19, 40) in the tumor tissue and enhanced suppressive activity toward proliferating T-cells (19). Overall, these results suggest that there is a significant link between stress, MDSC-induced immunosuppression, and cancer.

### Other Granulocytic Myeloid Cells

Other granulocytic cells such as basophils, eosinophils, and mast cells are mainly responsible for mediating allergic reactions, and neuroendocrine mediators can modulate their activity. To the best of our knowledge, the effects of stress on the activity of these cells, and how it affects cancer immunity is ripe for future research. However, their activity has been thoroughly studied in a non-cancer setting. It has been shown that stress hormones promote the production of T_H_2 cytokines (IL-4, IL-5, IL-10, IL-13) (12–14, 87). Importantly, IL-4 is an anti-inflammatory cytokine that stimulates the proliferation, activation, and migration of eosinophils and mast cells during allergic reaction responses (13, 14). For example, stress that was induced by exposure to pheromones released by footshock-stressed mice leads to an increase in IL-4 production and a decrease in IL-2 cytokine in affected mice (88).

Studies have found that healthy mice caged with tumor-bearing mice displayed elevated serum NE levels that correlated with increased production of pro-inflammatory cytokines (IL-4, IL-5) and increased lung inflammation (89). Specifically, IL-5 has been shown to serve as a chemoattractant for eosinophils in a variety of inflammatory diseases. Thus, stress-induced production of IL-5 may influence tumor immunity through eosinophilic recruitment into the tumor microenvironment.

## Lymphocytes: T-Cells, B Cells and NK Cells

### T-Cells

T-cells are part of the adaptive immune response and play a significant role in anti-tumoral immunity (90, 91). T_H_ cells are classified into T_H_1 cells that release interferon-gamma (IFN-g) and TNF-α, and T_H_2 cells that release IL-4, IL-5, and IL-13. Thus, when differentially activated, T-cells can contribute to the immune defense either through regulation of the immune response by recruiting other immune cells or by directly attacking infected or cancerous cells.

Activation of the SNS and HPA axis and subsequent release of glucocorticoids and catecholamines are known to suppress APCs and T_H_1 cells leading to a shift in T_H_2 cytokine production (12–14) and inhibiting the generation and activity of CTLs (12, 13). Animal models have consistently demonstrated a correlation between stress and T-cell immune dysregulation in cancer (15, 19–21, 92). For example, chronically restrained stressed mice displayed alterations in the T-lymphocyte subset distribution (less CD4+ T_H_), proliferation, cytokine secretion, and lymphocyte apoptosis (15). This same study also demonstrated that chronically stressed mice had worse overall survival and exhibited increased tumor growth (15).

In addition to alterations in T-cell proliferation and chemokine/cytokine secretion, chronic stress also leads to increased immunosuppression due to amplified activity of immune cells such as MDSCs and T-regulatory cells (19). Increased immunosuppression of MDSCs and T-regulatory cells can lead to tumor progression (93, 94). A study using sub-thermal temperature housing found that chronic stress in mice leads to reduced antitumor immune response, enhanced tumor growth, and increased metastasis (20). Also, a UV-induced squamous cell carcinoma model demonstrated that mice that exhibited anxiety-related behaviors displayed increased tumor burden, high corticosterone levels, decreased T-lymphocyte tumor infiltration, increased immunosuppressive activity and a prominent metastatic phenotype (21). Similarly, chronic psychosocially stressed mice exhibited increased immune suppressor cell activity toward proliferating T-cells and enhanced tumor size and weight (19). Additionally, surgically stressed mice displayed suppression of tumor-specific CD8+ T cells (95).

Clinical studies have also demonstrated that chronic levels of stress can lead to immune impairment in cancer patients (16, 17, 23, 37, 96). For example, studies that recruited surgically-treated breast cancer patients found that patients with high levels of stress exhibited reduced immune function indicated by lower ratios of T_H_1/T_H_2 cytokine production (96), and decreased T-cell activity (16, 17, 29). Similarly, a study of ovarian cancer patients found a correlation between depression and altered humoral-mediated immunity. These patients showed decreased activity of tumor-infiltrating lymphocytes (TILs), a predominant T_H_2 response, and lower T_H_1/ T_H_2 ratios in tumor-stimulated lymphocytes and peripheral blood cells (22, 23). Additionally, another study investigated the effects of depression on immunity in gastrointestinal cancer patients after the completion of the self-rating depression scale (SDS) and self-rating anxiety scale (SAS) questionnaires (18). This study found that patients in the depressed group had higher anxiety levels, poor social support, and a significant decline in lymphocyte count in comparison to non-depressed patients (18). As described in this section, the anti-tumoral activity of CTL and T_H_ cells are vital regulators of tumor immunity (90, 91) and thus could play a key role in chronic stress or depression/stress-induced tumor progression.

### B Cells

B-lymphocytes are antibody-producing cells that play an essential role in humoral immunity and immune homeostasis. B-cells exert their function either by direct interaction with surface receptors of other cells or through the release of immunomodulatory cytokines that affect the development and function of a variety of immune cells such as T-cells and DC (97). Antibodies released by B-cells can directly or indirectly influence tumor immunity. For example, B-cell-derived antibodies target cancer cells which then stimulate antigen presentation by DCs. Also, antibodies may indirectly contribute to tumor elimination by encouraging the cytotoxic activity of NK cells (98). B-cells are a key component of the tumor microenvironment, representing up to 60% of all tumor-infiltrating lymphocytes in breast and ovarian cancer patients (99, 100).

Recently, the role of B-cells in cancer immunity has gained more attention due to their possible association with carcinogenesis and progression (98). The ability of B cells to produce specific antibody isotypes may be influenced by the stress-induced shift in T_H_1 to T_H_2-mediated humoral immunity. At basal levels, T_H_1 cells express higher levels of AR binding sites than T_H_2, while β_2_-receptor activation differentially affects cytokine production. For example, T_H_1 activation of the β_2_-receptor suppresses the production of IFN- γ and consequently, the IFN-γ-dependent production of IgG2a by B cells (12). Furthermore, β_2_-receptor activation does not affect T_H_2 production of IL-4 nor subsequent IgG1 production by B cells (12).

Stress may also influence B-cell redistribution (21, 101, 102) and affect their survival (103). Notably, B-regulatory cells (Bregs) are capable of mediating pro-tumor immune responses by suppressing T-cells and anti-inflammatory cytokines (57). Recent studies have demonstrated that Bregs play an essential role in promoting tumor progression. For example, soluble factors secreted by tumor cells such as TNF-α, promote Bregs differentiation and enhances their activity (104). Tumor cells indirectly stimulate Bregs by recruiting T-cells that secrete IL-2,1 which induce Bregs differentiation (105). A recent phase II clinical trial in breast cancer patients found that perioperative administration of a β-antagonist and a COX2 inhibitor increased tumor-associated B-cells (106). This finding is especially important since tumor-infiltrating B-cells predict increased survival rates in cancer patients (107, 108). Furthermore, in a preclinical model of squamous cell carcinoma of the skin, it was found that mice exhibiting anxiety displayed T_H_1/T_H_2 cytokine shift and higher levels of Bregs which correlated with increased tumor burden (21). In essence, *in vitro*, and *in vivo* studies demonstrate that the stress-induced shift in T_H_1/T_H_2 immune responses may also modulate B-cell activity.

### NK Cells

NK cells are essential components of the innate immune system specialized in recognizing and attacking virus-infected, malignant, and tumor cells. T-cells and NK cells release IFN-γ and TNF, which leads to NK cell activation. NK cells differentiate between host and abnormal cells by recognition of MHC I receptors (109). Tumor cells often downregulate MHC I receptors to prevent recognition by CTLs but are still vulnerable to identification by NK cells (110).

Multiple studies have demonstrated that stress conditions lead to impairment of NK cell function. For example, acute swim stress and administration of EPI or β-agonists in rats, suppressed NK cell activity, demonstrating the involvement of adrenergic signaling in attenuation of immune cell function (111). Similarly, in a leukemia model, forced swim and administration of EPI or corticosterone inhibited NK activity and doubled rat mortality (25). These effects were blocked by the administration of a β-antagonist.

NK function impairment has also been investigated in many clinical studies. In a cohort of breast cancer patients, higher levels of stress were associated with decreased NK cell activity and IFN-γ (26). In another study, breast cancer patients with higher stress levels at baseline showed impaired natural killer cell cytotoxicity (NKCC) and decreased NK cell response when stimulated with IFN-γ (16). Furthermore, patients with elevated stress symptomatology had NK cells with altered membrane receptor expression (27). In ovarian cancer patients, distress was associated with poorer NKCC in TILs while social support was associated with higher NKCC in both TILs and circulating lymphocytes (22, 23). In addition, surgical stress impaired NKCC by suppressing IFN-γ secretion in colorectal cancer patients (112). These studies demonstrate that biobehavioral effects on immunity can also occur within the tumor microenvironment itself and impair multiple factors involved in NK cell activity. Currently, NK cells are considered a promising tool for cancer immunotherapies due to their role in immunosurveillance and malignant cell recognition. A recent review of the literature on this topic discusses several methods focused on enhancing the activity of NK cells for their potential use in cancer therapeutics (113).

## Stress, the Immune System, and Potential Therapeutic Targets

Currently, the incorporation of immunotherapeutic strategies into cancer treatment is steadily increasing. Researchers have a better understanding of the crosstalk between the tumor microenvironment and tumor cells, driving new therapies to target tumor-associated immune cells. For example, TAMs are considered a promising target for tumor therapy because of their capability to modulate different levels of tumorigenesis, such as inflammation, immunosuppression and metastatic potential of cancer cells (114). Preventing macrophage recruitment by pharmacological inhibition of chemoattractants, such as CCL2 (MCP-1), has proven successful in ovarian cancer (115), and pancreatic cancer (116). Macrophage depletion through induction of apoptosis, immunotoxin-conjugated monoclonal antibodies, and activating T cell-mediated recognition of TAMs are currently under study (117, 118). Zoledronic acid, a bisphosphonate currently used in metastatic breast cancer, eliminates MMP9-producing TAMs (119) and prolongs cancer patient survival (120). Another study showed that clodronate-mediated macrophage depletion reverts angiogenesis, tumor growth, and metastasis (121, 122). T cell-targeted therapies such as anti-PD-1 nivolumab (123) and anti-CTLA-4 ipilimumab (124) have shown great promise in clinical trials. Improved formulations of already established immunotherapies, such as recombinant IL-2 (125) and adoptive T cell therapy, for enhancing antitumor immune responses continue to demonstrate clinical benefit in various cancers (126, 127). The adverse systemic effects of chronic stress on immune cells, such as immunosuppression, might impair the positive therapeutic responses and benefits seen by current immunotherapies, highlighting the need to address SNS activation on tumor biology. For instance, ongoing stress can impair the efficacy of therapies based on immune stimulation (24, 128).

Several studies have proposed beta-adrenergic blockers to improve therapeutic outcomes in cancer patients due to the influence of the SNS in tumor biology (129). An existing body of work supports the harmful effects of adrenergic stimulation on cancer patients. β-adrenergic antagonists are suggested as a potential therapeutic strategy since they are widely available and used to treat conditions related to increased SNS activation, such as anxiety and hypertension. Recent clinical trials have begun studying the effects of including β-blockers in cancer patients' treatment regimens (NCT01308944, NCT02013492, NCT01847001). Interestingly, a clinical trial in MD Anderson Cancer Center (NCT01902966) combined relaxation and guided imagery sessions with propranolol to target both behavioral and molecular effects of SNS in cervical cancer. Multiple studies have shown that the use of beta-adrenergic antagonists improved the survival of cancer patients (130, 131), although others have reported no benefits with this treatment (132). A recent prospective pilot trial in epithelial ovarian cancer patients showed that chemotherapy in combination with propranolol improved depressive symptomatology, anxiety, quality of life, and decreased expression of pro-inflammatory genes (133). Although the known side effects of β-adrenergic blockers might limit their widespread use in cancer patients, preclinical, and clinical data support their use to abrogate the deleterious effects of chronic stress on cancer patients.

## Conclusion

Stress reduction programs have established social support networks for cancer patients while psychological therapy has also been utilized as a complementary approach to improve quality of life and clinical outcomes in cancer patients. Studies have shown that psychosocial interventions and psychological therapy in cancer patients have proven to be beneficial in reducing depression, anxiety, and improving quality of life (134, 135). However, some studies report mixed effects (136, 137). A combination of these strategies with immunotherapies and currently available chemotherapeutic treatments could significantly benefit patients under emotional stress and decrease the harmful consequences of neuroendocrine disruption. Further research is required to understand the complex interactions between neuroendocrine signaling pathways and tumor-associated immune cells. A comprehensive understanding of this process will facilitate the development and clinical application of targeted cancer immunotherapies.

## Author Contributions

CC-E, RL-C, and GA-P contributed to the conception and design of this review. CC-E and RL-C wrote the first draft of the manuscript. AA-A wrote sections of the manuscript. GA-P wrote sections of the manuscript and provided oversight. All authors read and approved the final manuscript.

### Conflict of Interest

The authors declare that the research was conducted in the absence of any commercial or financial relationships that could be construed as a potential conflict of interest.

## References

[1] Scanzano A Cosentino M. Adrenergic regulation of innate immunity: a review. Front Pharmacol. (2015) 6:586–518. 10.3389/fphar.2015.00171 26321956 PMC4534859

[2] Maddock C Pariante CM. How does stress affect you? An overview of stress, immunity, depression and disease. Epidemiol Psichiatr Soc. (2001) 10:153–62. 10.1017/S1121189X00005285 11787449

[3] Reiche EMV Nunes SOV Morimoto HK. Stress, depression, the immune system, and cancer. Lancet Oncol. (2004) 5:617–25. 10.1016/S1470-2045(04)01597-9 15465465

[4] Krizanova O Babula P Pacak K. *Stress* catecholaminergic system and cancer. Stress. (2016) 19:419–28. 10.1080/10253890.2016.1203415 27398826

[5] Tilan J Kitlinska J. Sympathetic neurotransmitters and tumor angiogenesis-link between stress and cancer progression. J Oncol. (2010) 2010:539706. 10.1155/2010/539706 20508839 PMC2874925

[6] Eng JW Kokolus KM Reed CB Hylander BL Ma WW Repasky EA. A nervous tumor microenvironment: the impact of adrenergic stress on cancer cells, immunosuppression, and immunotherapeutic response. Cancer Immunol Immunother. (2014) 63:1115–28. 10.1007/s00262-014-1617-9 25307152 PMC4325998

[7] Thaker PH Han LY Kamat AA Arevalo JM Takahashi R Lu C. Chronic stress promotes tumor growth and angiogenesis in a mouse model of ovarian carcinoma. Nat Med. (2006) 12:939–44. 10.1038/nm1447 16862152

[8] Nilsson MB Armaiz-Pena G Takahashi R Lin YG Trevino J Li Y. Stress hormones regulate interleukin-6 expression by human ovarian carcinoma cells through a Src-dependent mechanism. J Biol Chem. (2007) 282:29919–26. 10.1074/jbc.M611539200 17716980

[9] Sood AK Armaiz-Pena GN Halder J Nick AM Stone RL Hu W. Adrenergic modulation of focal adhesion kinase protects human ovarian cancer cells from anoikis. J Clin Invest. (2010) 120:1515–23. 10.1172/JCI40802 20389021 PMC2860925

[10] Armaiz-Pena GN Gonzalez-Villasana V Nagaraja AS Rodriguez-Aguayo C Sadaoui NC Stone RL. Adrenergic regulation of monocyte chemotactic protein 1 leads to enhanced macrophage recruitment and ovarian carcinoma growth. Oncotarget. (2015) 6:4266–73. 10.18632/oncotarget.2887 25738355 PMC4414188

[11] Cole SW Nagaraja AS Lutgendorf SK Green PA Sood AK. Sympathetic nervous system regulation of the tumour microenvironment. Nat Rev Cancer. (2015) 15:563–72. 10.1038/nrc3978 26299593 PMC4828959

[12] Sanders VM Baker RA Ramer-Quinn DS Kasprowicz DJ Fuchs BA Street NE. Differential expression of the beta2-adrenergic receptor by Th1 and Th2 clones: implications for cytokine production and B cell help. J Immunol. (1997) 158:4200–10. 9126981

[13] Elenkov IJ Chrousos GP. Stress hormones, Th1/Th2 patterns, pro/anti-inflammatory cytokines and susceptibility to disease. Trends Endocrinol Metab. (1999) 10:359–68. 10.1016/S1043-2760(99)00188-5 10511695

[14] Webster JI Tonelli L Sternberg EM. Neuroendocrine regulation of immunity. Annu Rev Immunol. (2002) 20:125–63. 10.1146/annurev.immunol.20.082401.104914 11861600

[15] Frick LR Arcos ML Rapanelli M Zappia MP Brocco M Mongini C. Chronic restraint stress impairs T-cell immunity and promotes tumor progression in mice. Stress. (2009) 12:134–43. 10.1080/10253890802137437 18609297

[16] Andersen BL Farrar WB Golden-Kreutz D Kutz LA MacCallum R Courtney ME. Stress and immune responses after surgical treatment for regional breast cancer. J Nat Cancer Instit. (1998) 90:36. 10.1093/jnci/90.1.30 9428780 PMC2743254

[17] Thornton LM Andersen BL Crespin TR Carson WE. Individual trajectories in stress covary with immunity during recovery from cancer diagnosis and treatments. Brain Behav Immun. (2007) 21:185–94. 10.1016/j.bbi.2006.06.007 16908118 PMC2151213

[18] Nan KJ Wei YC Zhou FL Li CL Sui CG Hui LY. Effects of depression on parameters of cell-mediated immunity in patients with digestive tract cancers. World J Gastroenterol. (2004) 10:268–72. 10.3748/wjg.v10.i2.268 14716837 PMC4717018

[19] Schmidt D Peterlik D Reber SO Lechner A Männel DN. Induction of suppressor cells and increased tumor growth following chronic psychosocial stress in male mice. PLoS ONE. (2016) 11:e0159059. 10.1371/journal.pone.0159059 27391954 PMC4938385

[20] Kokolus KM Capitano ML Lee CT Eng JW Waight JD Hylander BL. Baseline tumor growth and immune control in laboratory mice are significantly influenced by subthermoneutral housing temperature. Proc Natl Acad Sci USA. (2013) 110:20176–81. 10.1073/pnas.1304291110 24248371 PMC3864348

[21] Dhabhar FS Malarkey WB Neri E McEwen BS. Stress-induced redistribution of immune cells—from barracks to boulevards to battlefields: a tale of three hormones — curt richter award winner. Psychoneuroendocrinology. (2012) 37:1345–68. 10.1016/j.psyneuen.2012.05.008 22727761 PMC3412918

[22] Lutgendorf SK Sood AK Anderson B McGinn S Maiseri H Dao M. Social support, psychological distress, and natural killer cell activity in ovarian cancer. J Clin Oncol. (2005) 23:7105–13. 10.1200/JCO.2005.10.015 16192594

[23] Lutgendorf SK Lamkin DM DeGeest K Anderson B Dao M McGinn S. Depressed and anxious mood and T-cell cytokine expressing populations in ovarian cancer patients. Brain Behavior and Immunity. (2008) 22:890–900. 10.1016/j.bbi.2007.12.012 18276105 PMC2605940

[24] Nissen MD Sloan EK Mattarollo SR. β-adrenergic signaling impairs antitumor CD8 +T-cell responses to B-cell lymphoma immunotherapy. Cancer Immunol Res. (2018) 6:98–109. 10.1158/2326-6066.CIR-17-0401 29146881

[25] Inbar S Neeman E Avraham R Benish M Rosenne E Ben-Eliyahu S. Do stress responses promote leukemia progression? An animal study suggesting a role for epinephrine and prostaglandin-E2 through reduced NK activity. PLoS ONE. (2011) 6:e19246. 10.1371/journal.pone.0019246 21559428 PMC3084788

[26] Von Ah D Kang DH Carpenter JS. Stress, optimism, and social support: impact on immune responses in breast cancer. Res Nurs Health. (2007) 30:72–83. 10.1002/nur.20164 17243109

[27] Varker KA Terrell CE Welt M Suleiman S Thornton L Andersen BL Impaired natural killer cell lysis in breast cancer patients with high levels of psychological stress is associated with altered expression of killer immunoglobulin-like receptors. J Surg Res. (2007) 139:36–44. 10.1016/j.jss.2006.08.037 17292412 PMC1932802

[28] Lamkin DM Lutgendorf SK McGinn S Dao M Maiseri H DeGeest K. Positive psychosocial factors and NKT cells in ovarian cancer patients. Brain Behav Immun. (2008) 22:65–73. 10.1016/j.bbi.2007.06.005 17643954 PMC2964139

[29] Mohamed Gamal A Impact of stress on immune response of breast cancer women after mastectomy. AJNS. (2015) 4:182–8. 10.11648/j.ajns.20150404.16

[30] Matyszak MK Citterio S Rescigno M Ricciardi-Castagnoli P. Differential effects of corticosteroids during different stages of dendritic cell maturation. Eur J Immunol. (2000) 30:1233–42. 10.1002/(SICI)1521-4141(200004)30:4<1233::AID-IMMU1233>3.0.CO;2-F 10760813

[31] Piemonti L Monti P Allavena P Sironi M Soldini L Leone BE. Glucocorticoids affect human dendritic cell differentiation and maturation. J Immunol. (1999) 162:6473–81. 10352262

[32] Chamorro S García-Vallejo JJ Unger WW Fernandes RJ Bruijns SC Laban S. TLR triggering on tolerogenic dendritic cells results in TLR2 up-regulation and a reduced proinflammatory immune program. J Immunol. (2009) 183:2984–94. 10.4049/jimmunol.0801155 19648269

[33] Maestroni GJM. Dendritic cell migration controlled by 1b-adrenergic receptors. J Immunol. (2000) 165:6743–7. 10.4049/jimmunol.165.12.6743 11120793

[34] Maestroni GJM Mazzola P Langerhans cells β2-adrenoceptors: role in migration, cytokine production, Th priming and contact hypersensitivity. J Neuroimmunol. (2003) 144:91–9. 10.1016/j.jneuroim.2003.08.039 14597102

[35] Botta F Maestroni GJM. Adrenergic modulation of dendritic cell cancer vaccine in a mouse model: role of dendritic cell maturation. J Immunother. (2008) 31:263–70. 10.1097/CJI.0b013e318160995e 18317361

[36] Kokolus KM Spangler HM Povinelli BJ Farren MR Lee KP Repasky EA. Stressful presentations: mild cold stress in laboratory mice influences phenotype of dendritic cells in naïve and tumor-bearing mice. Front Immunol. (2014) 5:23. 10.3389/fimmu.2014.00023 24575090 PMC3918933

[37] Segerstrom SC Miller GE. Psychological stress and the human immune system: a meta-analytic study of 30 years of inquiry. Psychol Bull. (2004) 130:601–30. 10.1037/0033-2909.130.4.601 15250815 PMC1361287

[38] Jin J Wang X Wang Q Guo X Cao J Zhang X. Chronic psychological stress induces the accumulation of myeloid-derived suppressor cells in mice. PLoS ONE. (2013) 8:e74497. 10.1371/journal.pone.0074497 24058577 PMC3776856

[39] Mundy-Bosse BL Thornton LM Yang H-C Andersen BL Carson WE. Psychological stress is associated with altered levels of myeloid-derived suppressor cells in breast cancer patients. Cell Immunol. (2011) 270:80–7. 10.1016/j.cellimm.2011.04.003 21600570 PMC3129455

[40] Sloan EK Priceman SJ Cox BF Yu S Pimentel MA Tangkanangnukul V. The sympathetic nervous system induces a metastatic switch in primary breast cancer. Cancer Res. (2010) 70:7042–52. 10.1158/0008-5472.CAN-10-0522 20823155 PMC2940980

[41] Powell ND Tarr AJ Sheridan JF. Psychosocial stress and inflammation in cancer. Brain Behav Immun. (2013) 30:S41–S47. 10.1016/j.bbi.2012.06.015 22790082

[42] Sá-Rocha VM Sá-Rocha LC Palermo-Neto J. Variations in behavior, innate immunity and host resistance to B16F10 melanoma growth in mice that present social stable hierarchical ranks. Physiol Behav. (2006) 88:108–15. 10.1016/j.physbeh.2006.03.015 16647094

[43] Lin X Zheng W Liu J Zhang Y Qin H Wu H. Oxidative stress in malignant melanoma enhances tumor necrosis factor-alpha secretion of tumor-associated macrophages that promote cancer cell invasion. Antioxid Redox Signal. (2013) 19:1337–55. 10.1089/ars.2012.4617 23373752

[44] Qin J-F Jin F-J Li N Guan H-T Lan L Ni H. Adrenergic receptor β2 activation by stress promotes breast cancer progression through macrophages M2 polarization in tumor microenvironment. BMB Rep. (2015) 48:295–300. 10.5483/BMBRep.2015.48.5.008 25748171 PMC4578570

[45] Lamkin DM Ho HY Ong TH Kawanishi CK Stoffers VL Ahlawat N. β-adrenergic-stimulated macrophages: comprehensive localization in the M1-M2 spectrum. Brain Behav Immun. (2016) 57:338–46. 10.1016/j.bbi.2016.07.162 27485040 PMC5011037

[46] Black PH. Stress and the inflammatory response: A review of neurogenic inflammation. Brain Behav Immun. (2002) 16:622–53. 10.1016/S0889-1591(02)00021-1 12480495

[47] Szelényi J Kiss JP Vizi ES. Differential involvement of sympathetic nervous system and immune system in the modulation of TNF-alpha production by alpha2- and beta-adrenoceptors in mice. J Neuroimmunol. (2000) 103:34–40. 10.1016/s0165-5728(99)00234-9 10674987

[48] Van Miert A. Present concepts on the inflammatory modulators with special reference to cytokines. Vet Res Commun. (2002) 26:111–26. 10.1023/A:1014043601287 11924601

[49] Elenkov IJ Chrousos GP. Stress hormones, proinflammatory and antiinflammatory cytokines, and autoimmunity. Ann N Y Acad Sci. (2002) 966:290–303. 10.1111/j.1749-6632.2002.tb04229.x 12114286

[50] Lutgendorf SK Lamkin DM Jennings NB Arevalo JM Penedo F DeGeest K. Biobehavioral influences on matrix metalloproteinase expression in ovarian carcinoma. Clin Cancer Res. (2008) 14:6839–46. 10.1158/1078-0432.CCR-08-0230 18980978 PMC2716059

[51] Lamkin DM Srivastava S Bradshaw KP Bet JE Muy KB Wiese AM. C/EBPβ regulates the M2 transcriptome in β-adrenergic-stimulated macrophages. Brain Behav Immun. (2019) 80:839–48. 10.1016/j.bbi.2019.05.034 31132458 PMC6660400

[52] Bellinger DL Millar BA Perez S Carter J Wood C ThyagaRajan S. Sympathetic modulation of immunity: relevance to disease. Cell Immunol. (2008) 252:27–56. 10.1016/j.cellimm.2007.09.005 18308299 PMC3551630

[53] Reiche EMV Morimoto HK Nunes SMV. Stress and depression-induced immune dysfunction: implications for the development and progression of cancer. Int Rev Psychiatry. (2005) 17:515–27. 10.1080/02646830500382102 16401550

[54] Glaser R Kiecolt-Glaser JK. Stress-induced immune dysfunction: implications for health. Nat Rev Immunol. (2005) 5:243–51. 10.1038/nri1571 15738954

[55] Ben-Eliyahu S Yirmiya R Liebeskind JC Taylor AN Gale RP. Stress increases metastatic spread of a mammary tumor in rats: evidence for mediation by the immune system. Brain Behav Immun. (1991) 5:193–205. 10.1016/0889-1591(91)90016-4 1654166

[56] Lutgendorf S Cole S Costanzo E Bradley S Coffin J Jabbari S. Stress-related mediators stimulate vascular endothelial growth factor secretion by two ovarian cancer cell lines. Clin Cancer Res. (2003) 9:4514–21. 14555525

[57] Sarvaria A Madrigal JA Saudemont A. B cell regulation in cancer and anti-tumor immunity. Cell Mol Immunol. (2017) 14:662–74. 10.1038/cmi.2017.35 28626234 PMC5549607

[58] Stacker SA Williams SP Karnezis T Shayan R Fox SB Achen MG. Lymphangiogenesis and lymphatic vessel remodelling in cancer. Nat Rev Cancer. (2014) 14:159–72. 10.1038/nrc3677 24561443

[59] Le CP Nowell CJ Kim-Fuchs C Botteri E Hiller JG Ismail H. Chronic stress in mice remodels lymph vasculature to promote tumour cell dissemination. Nat Commun. (2016) 7:10634. 10.1038/ncomms10634 26925549 PMC4773495

[60] Murray PJ. Macrophage polarization. Annu Rev Physiol. (2017) 79:541–66. 10.1146/annurev-physiol-022516-034339 27813830

[61] Mantovani A Allavena P. The interaction of anticancer therapies with tumor-associated macrophages. J Exp Med. (2015) 212:435–45. 10.1084/jem.20150295 25753580 PMC4387285

[62] Ruffell B Affara NI Coussens LM. Differential macrophage programming in the tumor microenvironment. Trends Immunol. (2012) 33:119–26. 10.1016/j.it.2011.12.001 22277903 PMC3294003

[63] Qian BZ Pollard JW. Macrophage diversity enhances tumor progression and metastasis. Cell. (2010) 141:39–51. 10.1016/j.cell.2010.03.014 20371344 PMC4994190

[64] Bergers G Brekken R McMahon G Vu TH Itoh T Tamaki K. Matrix metalloproteinase-9 triggers the angiogenic switch during carcinogenesis. Nat Cell Biol. (2000) 2:737–44. 10.1038/35036374 11025665 PMC2852586

[65] Deryugina EI Quigley JP. Tumor angiogenesis: MMP-mediated induction of intravasation- and metastasis-sustaining neovasculature. Matrix Biol. (2015) 44–46, 94–112. 10.1016/j.matbio.2015.04.004 25912949 PMC5079283

[66] Jablonska-Trypuc A Matejczyk M Rosochacki S. Matrix metalloproteinases (MMPs), the main extracellular matrix (ECM) enzymes in collagen degradation, as a target for anticancer drugs. J Enzyme Inhib Med Chem. (2016) 31(Supp1. 1):177–83. 10.3109/14756366.2016.1161620 27028474

[67] Liu CY Xu JY Shi XY Huang W Ruan TY Xie P. M2-polarized tumor-associated macrophages promoted epithelial-mesenchymal transition in pancreatic cancer cells, partially through TLR4/IL-10 signaling pathway. Lab Invest. (2013) 93:844–54. 10.1038/labinvest.2013.69 23752129

[68] Fu XT Dai Z Song K Zhang ZJ Zhou ZJ Zhou SL. Macrophage-secreted IL-8 induces epithelial-mesenchymal transition in hepatocellular carcinoma cells by activating the JAK2/STAT3/Snail pathway. Int J Oncol. (2015) 46:587–96. 10.3892/ijo.2014.2761 25405790

[69] Bingle L Brown NJ Lewis CE. The role of tumour-associated macrophages in tumour progression: implications for new anticancer therapies. J Pathol. (2002) 196:254–65. 10.1002/path.1027 11857487

[70] Campbell MJ Tonlaar NY Garwood ER Huo D Moore DH Khramtsov AI. Proliferating macrophages associated with high grade, hormone receptor negative breast cancer and poor clinical outcome. Breast Cancer Res Treat. (2011) 128:703–11. 10.1007/s10549-010-1154-y 20842526 PMC4657137

[71] Komohara Y Hasita H Ohnishi K Fujiwara Y Suzu S Eto M. Macrophage infiltration and its prognostic relevance in clear cell renal cell carcinoma. Cancer Sci. (2011) 102:1424–31. 10.1111/j.1349-7006.2011.01945.x 21453387

[72] Sugimura K Miyata H Tanaka K Takahashi T Kurokawa Y Yamasaki M. High infiltration of tumor-associated macrophages is associated with a poor response to chemotherapy and poor prognosis of patients undergoing neoadjuvant chemotherapy for esophageal cancer. J Surg Oncol. (2015) 111:752–9. 10.1002/jso.23881 25752960

[73] Bower JE Shiao SL Sullivan P Lamkin DM Atienza R Mercado F. Prometastatic molecular profiles in breast tumors from socially isolated women. JNCI Cancer Spectr. (2018) 2:pky029. 10.1093/jncics/pky029 30057973 PMC6054021

[74] Cole SW Hawkley LC Arevalo JM Sung CY Rose RM Cacioppo JT. Social regulation of gene expression in human leukocytes. Genome Biol. (2007) 8:R189. 10.1186/gb-2007-8-9-r189 17854483 PMC2375027

[75] Vieira PL de Jong EC Wierenga EA Kapsenberg ML Kalinski P. Development of Th1-inducing capacity in myeloid dendritic cells requires environmental instruction. J Immunol. (2000) 164:4507–12. 10.4049/jimmunol.164.9.4507 10779751

[76] Amodio G Gregori S. Dendritic cells a double-edge sword in autoimmune responses. Front Immunol. (2012) 3:233. 10.3389/fimmu.2012.00233 22876246 PMC3410601

[77] Palucka K Banchereau J. Dendritic-cell-based therapeutic cancer vaccines. Immunity. (2013) 39:38–48. 10.1016/j.immuni.2013.07.004 23890062 PMC3788678

[78] Gabrilovich DI Chen HL Girgis KR Cunningham HT Meny GM Nadaf S. Production of vascular endothelial growth factor by human tumors inhibits the functional maturation of dendritic cells. Nat Med. (1996) 2:1096–103. 10.1038/nm1096-1096 8837607

[79] Stassi G Todaro M Zerilli M Ricci-Vitiani L Di Liberto D Patti M. Thyroid cancer resistance to chemotherapeutic drugs via autocrine production of interleukin-4 and interleukin-10. Cancer Res. (2003) 63:6784–90. 14583474

[80] Conticello C Pedini F Zeuner A Patti M Zerilli M Stassi G. IL-4 protects tumor cells from anti-CD95 and chemotherapeutic agents via up-regulation of antiapoptotic proteins. J Immunol. (2004) 172:5467–77. 10.4049/jimmunol.172.9.5467 15100288

[81] King C Mueller Hoenger R Malo Cleary M Murali-Krishna K Ahmed R King E. Interleukin-4 acts at the locus of the antigen-presenting dendritic cell to counter-regulate cytotoxic CD8+ T-cell responses. Nat Med. (2001) 7:206–14. 10.1038/84659 11175852

[82] Kim M-H Gorouhi F Ramirez S Granick JL Byrne BA Soulika AM Catecholamine stress alters neutrophil trafficking and impairs wound healing by β 2 -adrenergic receptor–mediated upregulation of IL-6. J Invest Dermatol. (2014) 134:809–17. 10.1038/jid.2013.415 24121404 PMC4013292

[83] Tsukamoto K Machida K. Effects of psychological stress on neutrophil phagocytosis and bactericidal activity in humans — a meta-analysis. Int J Psychophysiol. (2014) 91:67–72. 10.1016/j.ijpsycho.2013.12.001 24321824

[84] Liles WC Dale DC Klebanoff SJ. Glucocorticoids inhibit apoptosis of human neutrophils. Blood. (1995) 86:3181–8. 7579413

[85] Ko JS Zea AH Rini BI Ireland JL Elson P Cohen P. Sunitinib mediates reversal of myeloid-derived suppressor cell accumulation in renal cell carcinoma patients. Clin Cancer Res. (2009) 15:2148–57. 10.1158/1078-0432.CCR-08-1332 19276286

[86] Gabrilovich DI Nagaraj S. Myeloid-derived suppressor cells as regulators of the immune system. Nat Rev Immunol. (2009) 9:162–74. 10.1038/nri2506 19197294 PMC2828349

[87] Dhabhar FS. Stress-induced augmentation of immune function-the role of stress hormones, leukocyte trafficking, and cytokines. Brain Behav Immun. (2011) 16:785–98. 10.1016/S0889-1591(02)00036-3 12480507

[88] Moynihan JA Karp JD Cohen N Cocke R. Alterations in interleukin-4 and antibody production following pheromone exposure: role of glucocorticoids. J Neuroimmunol. (1994) 54:51–8. 10.1016/0165-5728(94)90230-5 7929803

[89] Hamasato EK de Lima APN de Oliveira APL Santos Franco dos AL, de Lima WT, Palermo-Neto J. Cohabitation with a sick partner increases allergic lung inflammatory response in mice. Brain Behav Immun. (2014) 42:109–17. 10.1016/j.bbi.2014.06.001 24929194

[90] Foss FM. Immunologic mechanisms of antitumor activity. Semin Oncol. (2002) 29:5–11. 10.1053/sonc.2002.33076 12068382

[91] Ostrand-Rosenberg S. CD4 T lymphocytes: a critical component of antitumor immunity. LCNV. (2005) 23:413–9. 10.1081/CNV-200067428 16193641

[92] Reber SO Birkeneder L Veenema AH Obermeier F Falk W Straub RH. Adrenal insufficiency and colonic inflammation after a novel chronic psycho-social stress paradigm in mice: implications and mechanisms. Endocrinology. (2007) 148:670–82. 10.1210/en.2006-0983 17110427

[93] Gabrilovich DI. Myeloid-derived suppressor cells. Cancer Immunol Res. (2017) 5:3–8. 10.1158/2326-6066.CIR-16-0297 28052991 PMC5426480

[94] Ostrand-Rosenberg S Sinha P Beury DW Clements VK. Cross-talk between myeloid-derived suppressor cells (MDSC), macrophages, and dendritic cells enhances tumor-induced immune suppression. Semin Cancer Biol. (2012) 22:275–81. 10.1016/j.semcancer.2012.01.011 22313874 PMC3701942

[95] Ananth AA Tai L-H Lansdell C Alkayyal AA Baxter KE Angka L Surgical stress abrogates pre-existing protective T cell mediated anti-tumor immunity leading to postoperative cancer recurrence. PLoS ONE. (2016) 11:e0155947 10.1371/journal.pone.0155947 27196057 PMC4873120

[96] Blomberg BB Alvarez JP Diaz A Romero MG Lechner SC Carver CS. Psychosocial adaptation and cellular immunity in breast cancer patients in the weeks after surgery: an exploratory study. J Psychosomat Res. (2009) 67:369–76. 10.1016/j.jpsychores.2009.05.016 19837199 PMC2764537

[97] Harris DP Haynes L Sayles PC Duso DK Eaton SM Lepak NM. Reciprocal regulation of polarized cytokine production by effector B and T cells. Nat Immunol. (2000) 1:475–82. 10.1038/82717 11101868

[98] Yuen GJ Demissie E Pillai S. B lymphocytes and cancer: a love–hate relationship. Trends Cancer. (2016) 2:747–57. 10.1016/j.trecan.2016.10.010 28626801 PMC5472356

[99] Coronella-Wood JA Hersh EM. Naturally occurring B-cell responses to breast cancer. Cancer Immunol Immunother. (2003) 52:715–38. 10.1007/s00262-003-0409-4 12920480 PMC11033039

[100] Nelson BH. CD20+ B cells: the other tumor-infiltrating lymphocytes. J Immunol. (2010) 185:4977–82. 10.4049/jimmunol.1001323 20962266

[101] Dhabhar FS McEwen BS. Acute stress enhances while chronic stress suppresses cell-mediated immunityin vivo: a potential role for leukocyte trafficking. Brain Behav Immun. (1997) 11:286–306. 10.1006/brbi.1997.0508 9512816

[102] Engler H Dawils L Hoves S Kurth S Stevenson JR Schauenstein K. Effects of social stress on blood leukocyte distribution: the role of α- and β-adrenergic mechanisms. J Neuroimmunol. (2004) 156:153–62. 10.1016/j.jneuroim.2004.08.005 15465606

[103] Trottier MD Newsted MM King LE Fraker PJ. Natural glucocorticoids induce expansion of all developmental stages of murine bone marrow granulocytes without inhibiting function. Proc Natl Acad Sci USA. (2008) 105:2028–33. 10.1073/pnas.0712003105 18250324 PMC2538876

[104] Schioppa T Moore R Thompson RG Rosser EC Kulbe H Nedospasov S. B regulatory cells and the tumor-promoting actions of TNF-α during squamous carcinogenesis. Proc Natl Acad Sci USA. (2011) 108:10662–7. 10.1073/pnas.1100994108 21670304 PMC3127875

[105] Lindner S Dahlke K Sontheimer K Hagn M Kaltenmeier C Barth TF. Interleukin 21-induced granzyme B-expressing B cells infiltrate tumors and regulate T cells. Cancer Res. (2013) 73:2468–79. 10.1158/0008-5472.CAN-12-3450 23384943

[106] Shaashua L Shabat-Simon M Haldar R Matzner P Zmora O Shabtai M. Perioperative COX-2 and β-adrenergic blockade improves metastatic biomarkers in breast cancer patients in a phase-II randomized trial. Clin Cancer Res. (2017) 23:4651–61. 10.1158/1078-0432.CCR-17-0152 28490464 PMC5559335

[107] Schmidt M Böhm D von Törne C Steiner E Puhl A Pilch H. The humoral immune system has a key prognostic impact in node-negative breast cancer. Cancer Res. (2008) 68:5405–13. 10.1158/0008-5472.CAN-07-5206 18593943

[108] Wouters MCA Nelson BH. Prognostic significance of tumor-infiltrating B cells and plasma cells in human cancer. Clin Cancer Res. (2018) 24:6125–35. 10.1158/1078-0432.CCR-18-1481 30049748

[109] Dahlberg CIM Sarhan D Chrobok M Duru AD Alici E. Natural killer cell-based therapies targeting cancer: possible strategies to gain and sustain anti-tumor activity. Front Immunol. (2015) 6:605. 10.3389/fimmu.2015.00605 26648934 PMC4663254

[110] Mandal A Viswanathan C. Natural killer cells: In health and disease. Hematol Oncol Stem Cell Ther. (2015) 8:47–55. 10.1016/j.hemonc.2014.11.006 25571788

[111] Ben-Eliyahu S Shakhar G Page GG Stefanski V Shakhar K Suppression of NK cell activity and of resistance to metastasis by stress: a role for adrenal catecholamines and ß-adrenoceptors. Neuro Immuno Modul. (2000) 8:154–64. 10.1159/000054276 11124582

[112] Angka L Martel AB Kilgour M Jeong A Sadiq M de Souza CT. Natural killer cell IFNγ secretion is profoundly suppressed following colorectal cancer surgery. Ann Surg Oncol. (2018) 25:3747–54. 10.1245/s10434-018-6691-3 30187278

[113] Lowry LE Zehring WA. Potentiation of natural killer cells for cancer immunotherapy: a review of literature. Front Immunol. (2017) 8:1061. 10.3389/fimmu.2017.01061 28919894 PMC5585139

[114] Tang X Mo C Wang Y Wei D Xiao H. Anti-tumour strategies aiming to target tumour-associated macrophages. Immunology. (2013) 138:93–104. 10.1111/imm.12023 23113570 PMC3575762

[115] Lopez-Guerrero JA Romero I Poveda A. Trabectedin therapy as an emerging treatment strategy for recurrent platinum-sensitive ovarian cancer. Chin J Cancer. (2015) 34:41–9. 10.5732/cjc.014.10278 25556617 PMC4302088

[116] Nywening TM Wang-Gillam A Sanford DE Belt BA Panni RZ Cusworth BM. Targeting tumour-associated macrophages with CCR2 inhibition in combination with FOLFIRINOX in patients with borderline resectable and locally advanced pancreatic cancer: a single-centre, open-label, dose-finding, non-randomised, phase 1b trial. Lancet Oncol. (2016) 17:651–62. 10.1016/S1470-2045(16)00078-4 27055731 PMC5407285

[117] Germano G Frapolli R Belgiovine C Anselmo A Pesce S Liguori M. Role of macrophage targeting in the antitumor activity of trabectedin. Cancer Cell. (2013) 23:249–62. 10.1016/j.ccr.2013.01.008 23410977

[118] Nagai T Tanaka M Tsuneyoshi Y Xu B Michie SA Hasui K. Targeting tumor-associated macrophages in an experimental glioma model with a recombinant immunotoxin to folate receptor beta. Cancer Immunol Immunother. (2009) 58:1577–86. 10.1007/s00262-009-0667-x 19238383 PMC11030051

[119] Giraudo E Inoue M Hanahan D. An amino-bisphosphonate targets MMP-9–expressing macrophages and angiogenesis to impair cervical carcinogenesis. J Clin Investig. (2004) 114:623–33. 10.1172/JCI200422087 15343380 PMC514591

[120] Eidtmann H de Boer R Bundred N Llombart-Cussac A Davidson N Neven P. Efficacy of zoledronic acid in postmenopausal women with early breast cancer receiving adjuvant letrozole: 36-month results of the ZO-FAST Study. Ann Oncol. (2010) 21:2188–94. 10.1093/annonc/mdq217 20444845

[121] Zeisberger SM Odermatt B Marty C Zehnder-Fjallman AH Ballmer-Hofer K Schwendener RA. Clodronate-liposome-mediated depletion of tumour-associated macrophages: a new and highly effective antiangiogenic therapy approach. Br J Cancer. (2006) 95:272–81. 10.1038/sj.bjc.6603240 16832418 PMC2360657

[122] Hiraoka K Zenmyo M Watari K Iguchi H Fotovati A Kimura YN. Inhibition of bone and muscle metastases of lung cancer cells by a decrease in the number of monocytes/macrophages. Cancer Sci. (2008) 99:1595–602. 10.1111/j.1349-7006.2008.00880.x 18754872 PMC11158597

[123] Motzer RJ Rini BI McDermott DF Redman BG Kuzel TM Harrison MR. Nivolumab for metastatic renal cell carcinoma: results of a randomized phase II trial. J Clin Oncol. (2015) 33:1430–7. 10.1200/JCO.2014.59.0703 25452452 PMC4806782

[124] Hamid O Schmidt H Nissan A Ridolfi L Aamdal S Hansson J. A prospective phase II trial exploring the association between tumor microenvironment and clinical activity of ipilimumab in advanced melanoma. J Transl Med. (2011) 9:204. 10.1186/1479-5876-9-204 22123319 PMC3239318

[125] Jiang T Zhou C Ren S. Role of IL-2 in cancer immunotherapy. Oncoimmunology. (2016) 5:e1163462. 10.1080/2162402X.2016.1163462 27471638 PMC4938354

[126] Turtle CJ Hanafi LA Berger C Gooley TA Cherian S Hudecek M. CD19 CAR-T cells of defined CD4+:CD8+ composition in adult B cell ALL patients. J Clin Invest. (2016) 126:2123–38. 10.1172/JCI85309 27111235 PMC4887159

[127] Ramos CA Savoldo B Torrano V Ballard B Zhang H Dakhova O. Clinical responses with T lymphocytes targeting malignancy-associated kappa light chains. J Clin Invest. (2016) 126:2588–96. 10.1172/JCI86000 27270177 PMC4922690

[128] Levi B Matzner P Goldfarb Y Sorski L Shaashua L Melamed R. Stress impairs the efficacy of immune stimulation by CpG-C: potential neuroendocrine mediating mechanisms and significance to tumor metastasis and the perioperative period. Brain Behav Immun. (2016) 56:209–20. 10.1016/j.bbi.2016.02.025 26944000 PMC4917466

[129] Cole SW Sood AK. Molecular pathways: beta-adrenergic signaling in cancer. Clin Cancer Res. (2012) 18:1201–6. 10.1158/1078-0432.CCR-11-0641 22186256 PMC3294063

[130] Watkins JL Thaker PH Nick AM Ramondetta LM Kumar S Urbauer DL. Clinical impact of selective and nonselective beta-blockers on survival in patients with ovarian cancer. Cancer. (2015) 121:3444–51. 10.1002/cncr.29392 26301456 PMC4575637

[131] Diaz ES Karlan BY Li AJ. Impact of beta blockers on epithelial ovarian cancer survival. Gynecol Oncol. (2012) 127:375–8. 10.1016/j.ygyno.2012.07.102 22819786

[132] Shah SM Carey IM Owen CG Harris T Dewilde S Cook DG. Does beta-adrenoceptor blocker therapy improve cancer survival? Findings from a population-based retrospective cohort study. Br J Clin Pharmacol. (2011) 72:157–61. 10.1111/j.1365-2125.2011.03980.x 21453301 PMC3141198

[133] Ramondetta LM Hu W Thaker PH Urbauer DL Chisholm GB Westin SN. Prospective pilot trial with combination of propranolol with chemotherapy in patients with epithelial ovarian cancer and evaluation on circulating immune cell gene expression. Gynecol Oncol. (2019) 154:524–30. 10.1016/j.ygyno.2019.07.004 31353053 PMC6867685

[134] Xiao F Song X Chen Q Dai Y Xu R Qiu C. Effectiveness of psychological interventions on depression in patients after breast cancer surgery: a meta-analysis of randomized controlled trials. Clin Breast Cancer. (2016) 17:171–9. 10.1016/j.clbc.2016.11.003 28040415

[135] Guo Z Tang H Li H Tan S Feng K Huang Y. The benefits of psychosocial interventions for cancer patients undergoing radiotherapy. Health Qual Life Outcomes. (2013) 11:121. 10.1186/1477-7525-11-121 23866850 PMC3721996

[136] Boesen EH Karlsen R Christensen J Paaschburg B Nielsen D Bloch IS. Psychosocial group intervention for patients with primary breast cancer: a randomised trial. Eur J Cancer. (2011) 47:1363–72. 10.1016/j.ejca.2011.01.002 21458989

[137] Rush SE Sharma M. Mindfulness-based stress reduction as a stress management intervention for cancer care: a systematic review. J Evid Based Complementary Altern Med. (2016) 22:348–60. 10.4324/9780203862568 27489233 PMC5871193

